# Associations between COVID-19 pandemic experiences and suicidal ideation among adolescents: the mediating role of life satisfaction, subjective wellbeing, and anxiety

**DOI:** 10.3389/fpubh.2026.1834645

**Published:** 2026-08-05

**Authors:** Helena Jeriček Klanšček, Petra Mikolič Brence, Carolina Blanco Calvo, Saška Roškar, Špela Selak, Matej Vinko

**Affiliations:** 1 National Institute of Public Health, Ljubljana, Slovenia; 2 Hospital Universitario Doctor Peset, Valencia, Spain

**Keywords:** adolescent, anxiety, COVID-19, life satisfaction, suicide ideation, wellbeing

## Abstract

**Introduction:**

The aim of this study was to examine associations between adolescents’ experiences during the COVID-19 pandemic and life satisfaction, subjective wellbeing, anxiety, and suicidal ideation.

**Methods:**

Data were drawn from the 2022 cross-national Health Behavior in School-aged Children study conducted in Slovenia, using a representative sample of 8,638 adolescents aged 11, 13, 15, and 17 years (51.7% girls). Adolescents reported on their experiences during the COVID-19 pandemic (hospitalization of a family member and perceived changes in relationships, school performance, future expectations, and family finances), life satisfaction, subjective wellbeing, anxiety, and suicidal ideation. A structural equation model (SEM) was tested, with pandemic-related experiences as predictors, mental health indicators as intervening variables, and suicidal ideation as the outcome.

**Results:**

Suicidal ideation was reported by 16.7% of adolescents. The model explained 45.4% of the variance in suicidal ideation. Perceived impacts of the pandemic on family relationships and future expectations, as well as the hospitalization of a family member, were associated with suicidal ideation both directly and indirectly through all three mental health indicators. The perceived impact on friendships was associated with suicidal ideation directly and indirectly via life satisfaction, whereas perceived changes in academic performance were associated with suicidal ideation both directly and indirectly via subjective wellbeing and anxiety. Pandemic-related changes in family relationships showed the strongest overall association with suicidal ideation.

**Conclusion:**

Pandemic-related family stressors and disruptions to future expectations were associated with suicidal ideation both directly and indirectly through poorer mental health indicators. These findings suggest the importance of integrated family- and school-based approaches to supporting adolescent mental health during periods of major societal disruption. Strengthening family support systems, early identification of anxiety symptoms, and school-based prevention programs may help mitigate adverse mental health outcomes among adolescents. The findings may also inform preparedness planning for future public health emergencies.

## 1 Introduction

Over the past two decades, adolescent mental health has become an increasing global public health concern. Emerging evidence suggests that the deterioration in young people’s mental health cannot be understood solely through individual vulnerability but must be viewed within the broader context of converging societal megatrends—including climate change, economic insecurity, widening social inequalities, digital transformation, geopolitical instability, and global health crises—that collectively shape developmental environments and opportunities (1). The COVID-19 pandemic represented one of the most profound manifestations of these interacting challenges, exposing and amplifying existing vulnerabilities while disrupting key developmental contexts such as family life, education, peer relationships, and future expectations (1).

Among all age groups, adolescents and young adults are among those most at risk of mental health conditions, since adolescence is considered a developmental period of increased exposure to social stressors and heightened stress reactivity (2), which has been shown to be related to suicidality (3). It is estimated that 1 in 5 adolescents in the WHO European Region experience mental health conditions (4), which underscores the importance of early interventions (5). The prevalence of mental health symptoms such as anxiety and depression, as well as suicidal ideation, in children and adolescents appears to have increased over the course of the pandemic (6–10). In this context, with a growing number of young people reporting poorer mental health and suicidal ideation, it is important to acknowledge how the pandemic has exacerbated the latter.

Research indicates that multiple contextual and relational factors influence adolescents’ mental health and suicidality. Family economic hardship, academic disruptions, family conflict, and social isolation have shown direct associations with higher levels of depression, anxiety, and suicidal ideation, suggesting that these stressors act as immediate psychosocial risks (11–13). In contrast, illness or hospitalization of family members and pessimistic future expectations primarily exert indirect effects, increasing vulnerability through mediating mechanisms such as heightened anxiety, psychological distress, and reduced wellbeing (14). Similarly, weakened family functioning often affects mental health indirectly by lowering life satisfaction and perceived emotional security, which in turn elevate the likelihood of suicidal thoughts (13). At the same time, peer and friend support consistently emerge as crucial protective factors. Strong peer connectedness shows direct links with lower depression, stress anxiety, and suicidality (13, 15–17). Collectively, these findings emphasize that adolescents’ vulnerability to poor mental health and suicidality during the pandemic arises from an interplay of direct stress exposure and indirect processes mediated by emotional wellbeing and social connectedness.

Suicidal thoughts and behaviors are a major but preventable public health concern and include suicidal ideation (i.e., thoughts about taking one’s own life), making a suicide plan, and attempting suicide. Suicidal ideation usually begins in adolescence and is observed in both clinical and community populations (18). In fact, suicide is the second leading cause of death worldwide among individuals aged 10–24 years (19) and the third leading cause of death among 15–29-year-olds globally (20). Adolescents who reported experiencing suicidal thoughts at the age of 15 were more likely, by age 30, to suffer from anxiety and depression and to function significantly less well as young adults (general social and occupational functioning) (21). Being a bully, loneliness, multiple health complaints, and depression are significant risk factors for suicidal ideation, whereas family support serves as a protective factor (18). Recent umbrella reviews by Richardson et al. (22) and McEvoy et al. (23) indicate that adolescent suicidality and self-harm are influenced by multiple interacting risk factors across individual, family, and social contexts. Both reviews identified mental health disorders, previous self-harm, bullying victimization, childhood adversity, family dysfunction, and social isolation as consistent predictors of increased risk, while supportive family and peer relationships emerged as important protective factors. Together, the findings suggest that youth suicidality is shaped by a complex socio-ecological process.

The problem of suicide among young people has raised concern, as suicidal ideation, self-harm, and attempted suicide have increased internationally in this population over the last decade, and the COVID-19 pandemic may have had the potential to worsen these trends (24). Ferreira et al. (25) found an increase in self-harm and suicidality among adolescents during the pandemic with girls being particularly affected. The observed increase was associated with social isolation, excessive screen time, reduced access to education and healthcare, and increased family or financial stress.

Following the COVID-19 outbreak, early evidence suggested that pandemic-related difficulties did not immediately lead to increased suicide rates (26, 27). However, as the pandemic and its countermeasures (e.g., social distancing, quarantine) persisted for several years, studies began reporting higher rates of pediatric emergency department visits for suicide-related behaviors, with mental health problems showing strong positive associations with suicidal ideation and attempts (14, 24, 28–30). A large population-based longitudinal cohort study from Germany identified distinct mental health trajectories (31). While most adolescents remained resilient during the pandemic, a sizable number (10–18%) experienced either internalizing or externalizing problems (31). Health-related quality of life was found to be significantly associated with class membership, personal resources, family climate and social support.

To our knowledge, few studies have concurrently examined adolescents’ experiences of COVID-19-related measures, mental health indicators, and suicidal ideation within a single analytical framework, and none have examined the complexity of their interrelationships in the manner proposed here. To address this gap, the present study draws on two complementary frameworks—the Conservation of Resources (COR) Theory (32) and the Dual-Factor Model of Mental Health (DFM) (33)—to capture the multidimensional nature of these associations. COR theory posits that individuals strive to obtain, maintain, and protect valued resources such as emotional support, social connections, and a sense of stability. Stress arises when these resources are lost or threatened, whereas resource gains foster resilience and recovery. During the pandemic, adolescents faced various forms of resource loss, including strained family relationships, disrupted schooling, and uncertainty about the future, while some experienced gains, such as strengthened family bonds or improved coping. The DFM complements this perspective by conceptualizing mental health as comprising two interrelated dimensions—positive functioning (e.g., life satisfaction and wellbeing) and psychological distress (e.g., anxiety). Together, these frameworks suggest that changes across key life domains shape adolescents’ mental health through both resource loss and resource gain, which may in turn influence suicidal ideation. Accordingly, this study examines how adolescents perceived changes in pandemic-affected domains—family member hospitalization, family and peer relationships, school performance, future expectations, and family finances—relate to positive and negative aspects of mental health and to suicidal ideation.

The present study adds to the knowledge base on the associations between experiences during the COVID-19 pandemic, mental health measures, and suicidal behavior among adolescents. The aim of the present study was to examine associations between adolescents’ experiences of COVID-19 pandemic measures (hospitalization of a family member, family relationships, friendships, school performance, future expectations, and family finances) and suicidal ideation, and to investigate the mediating role of life satisfaction, subjective wellbeing, and anxiety in Slovenia, a country with an average suicide rate of 18 per 100,000 (34) which is well above the EU average (35). From a public health perspective, understanding these associations may help identify key psychosocial targets for prevention efforts and inform preparedness planning for future public health emergencies. Such evidence is also essential for translating global mental health commitments into effective national policies by strengthening cross-sectoral approaches, addressing social determinants of mental health, and integrating youth mental health within broader public health strategies (36).

## 2 Materials and methods

### 2.1 Procedure and participants

The Health Behavior in School-aged Children (HBSC) study is a World Health Organization collaborative cross-national study that collects self-reported data on the health and wellbeing of adolescents (37). In this study, data from the 2022 HBSC study in Slovenia were used. A nationally representative sample comprised 8,638 students aged 11, 13, 15, and 17 years (51.7% girls), who completed online questionnaires in classrooms. The sampling unit was the school class. Information on student enrollment for the 2021/2022 school year was obtained from the Ministry of Education. The data included enrollment in the 6th and 8th grades of all primary schools in Slovenia (*N* = 489) and enrollment in the 1st and 3rd grades of all secondary schools (*N* = 154). Of the secondary schools, 80 offered general upper secondary education (gymnasium programs), 86 vocational secondary education programs, 39 lower vocational education programs, and 106 technical or other professional secondary education programs.

The enrollment data served as the sampling frame and formed the basis for constructing a representative sample. A two-stage stratified sampling design was employed. In the first stage, primary and secondary schools were selected. In the second stage, secondary schools were stratified according to the educational program offered (general upper secondary education, technical or other professional secondary education, vocational secondary education, and lower vocational education). The final response rate was 86.7%.

During data preparation, questionnaires with more than 50% missing responses were excluded from the dataset. The remaining data were processed according to the international HBSC protocol and submitted to the HBSC International Data Bank in Norway, where data from all participating countries underwent further quality control procedures based on internationally agreed standards. Additional records were excluded if respondents did not meet the required age criteria or if essential information was missing. In total, 310 records were removed because information on sex or grade was missing, or because the respondent’s age was outside the required range or inconsistent with the reported grade level.

The final analytical sample comprised 8,638 adolescents and served as the basis for all analyses conducted for the 2022 survey cycle. The sample included 2,082 students in Grade 6 (approximately 11 years old), 2,089 students in Grade 8 (approximately 13 years old), 2,151 students in the first year of secondary school (approximately 15 years old), and 2,309 students in the third year of secondary school (approximately 17 years old).

As the final sample distribution differed slightly from the national enrollment structure, post-stratification weights were applied. For primary school students, weighting was performed by sex within each grade (Grades 6 and 8). For secondary school students, weighting was performed within each grade level (first and third year of secondary school) according to educational program close and sex.

Informed consent was obtained from the parents of the adolescents involved in the study. Data collection was conducted between January 24 and February 18, 2022.

The study was approved by the National Medical Ethics Committee of the Republic of Slovenia (No. 0120–579/2021/6) and was conducted in accordance with the ethical principles of the World Medical Association Declaration of Helsinki and the recommendations of the International Committee of Medical Journal Editors (ICMJE).

Participation was voluntary and anonymous. Written informed consent was obtained from parents or legal guardians, and adolescents provided their assent prior to participation. No personally identifiable information was collected. The final weighted sample included 51.0% girls, with age distribution as follows: 23.8% 11-year-olds, 24.1% 13-year-olds, 25.3% 15-year-olds, and 26.9% 17-year-olds.

### 2.2 Measures

The measures used in this study were derived from the international HBSC questionnaire and protocol and represent established and validated instruments widely used in adolescent health research.

#### 2.2.1 Suicidal ideation

Suicidal ideation: Adolescents were asked whether they had seriously considered attempting suicide in the past 12 months (forced-choice response options: yes/no).

#### 2.2.2 Mental health measures

##### 2.2.2.1 Life satisfaction

Adolescents rated their life satisfaction on a scale from 0 (worst possible life) to 10 (best possible life) with wording suitable for children as young as 11 years old (38). Respondents ticked the number next to the step that best describes the position on the ladder where they feel they stand at the moment. The measure has extensive evidence of validity and reliability (39, 40). Mean scores were used for analysis.

##### 2.2.2.2 Subjective wellbeing

Subjective wellbeing was measured with the WHO-5 Wellbeing Index (41), assessing five positive states (e.g., feeling cheerful, calm, and vigorous) on a 6-point Likert scale. Scores were converted to a 0–100 range, with higher scores indicating better wellbeing. The WHO-5 Wellbeing Index is one of the most widely used brief measures of subjective wellbeing and has demonstrated good validity, reliability, and cross-national applicability (42). The internal consistency measured by Cronbach’s alpha in this study was *α* = 0.87.

##### 2.2.2.3 Anxiety symptoms

Anxiety symptoms were assessed using the Generalized Anxiety Disorder Scale (GAD-7) (43), a widely used and validated instrument that has been translated and applied internationally, including in Slovenia (44). GAD-7 rates anxiety symptoms on a 4-point Likert scale (0–3). This scale comprises seven items referring to the past 2 weeks. For example, participants were asked, “How often in the past 2 weeks have you been bothered by feeling nervous, anxious, or on edge?” (response options: not at all; several days; more than half the days; nearly every day). Total scores (0–21) reflect the severity of generalized anxiety disorder, with higher scores indicating greater symptom severity. Cronbach’s alpha in the present study was *α* = 0.90.

#### 2.2.3 COVID-19 pandemic experiences

##### 2.2.3.1 Hospitalization of a close family member for COVID-19

Adolescents reported whether a close family member had been hospitalized due to COVID-19 (forced-choice response options: yes/no).

##### 2.2.3.2 Self-perceived impact of the COVID-19 pandemic measures

The HBSC questionnaire included a set of 10 items assessing adolescents’ perceptions of how the COVID-19 pandemic affected their lives. It was developed by the HBSC network and uses a five-point Likert-type response format. Likert-like response format ranging from “very negative” (1) to “very positive” (5) with a neutral midpoint (3 = “neither positive nor negative”) to capture both the direction and intensity of adolescents’ perceptions of the impact of COVID-19 on life in general, overall health and mental health, relationships with family and friends, school performance, physical activity, eating behaviors, future expectations and family finances (45). For the purposes of this study, we focused on five domains most relevant to the literature on adolescent suicidality: family relationships, friendships, school performance, future expectations, and family finances. Each domain was analyzed as a separate variable to capture its unique association with adolescent mental health and suicidal ideation during the pandemic (37).

### 2.3 Statistical analysis

We examined descriptive statistics (means, standard deviations, and proportions) and bivariate relationships among the variables: correlations for continuous variables, independent samples *t*-tests for associations between continuous and dichotomous variables, and chi-square tests for associations between dichotomous variables. Effect sizes were evaluated using Cohen’s d and Cramer’s V, and interpreted according to standard thresholds where *d* ≈ 0.20 constitutes a small effect, 0.50 a medium effect, and 0.80 a large effect (46). Normality of continuous variables was assessed using Kolmogorov–Smirnov and Shapiro–Wilk tests; due to violations of normality, non-parametric Mann–Whitney U tests were conducted. Because these yielded identical significance (*p* < 0.001), independent samples *t*-tests are reported for ease of interpretation. Missing data on exogenous variables were handled using listwise deletion, while missing data were handled using the pairwise covariance coverage approach.

We performed structural equation modeling (SEM) using Mplus version 8.8 (47) with WLSMV estimation, and model fit was assessed with the Root Mean Square Error of Approximation (RMSEA), Comparative Fit Index (CFI), Tucker–Lewis Index (TLI), and Standardized Root Mean Square Residual (SRMR). To adjust for the stratified sampling design, post-stratification survey weights were applied to the primary SEM analyses. Prior to specifying the structural model, we conducted a Confirmatory Factor Analysis (CFA) with Maximum Likelihood estimation to verify the one-factor measurement models for the latent mediating variables. Both models demonstrated excellent fit (WHO-5: CFI = 0.987, TLI = 0.974, SRMR = 0.016; GAD-7: CFI = 0.987, TLI = 0.980, SRMR = 0.016). The WLSMV estimator was utilized because it is robust for models with categorical outcomes (suicidal ideation) and non-normally distributed continuous data. Specific indirect effects were formally evaluated to examine indirect associations consistent with the proposed mediation model (48, 49). No post-hoc model modifications were applied to the structural model.

#### 2.3.1 The model

**Figure 1:**
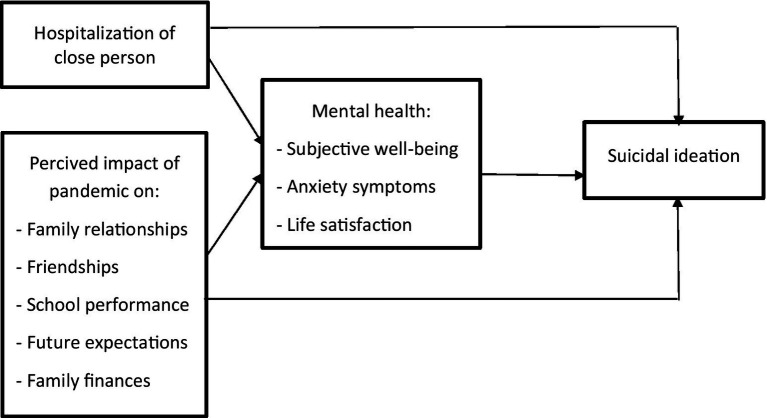
The proposed model of linking adolescents’ experience of COVID-19 pandemic measures to subjective wellbeing, anxiety symptoms, life satisfaction, and suicidal ideation.

The conceptual model of the associations between pandemic-related experiences, mental health indicators, and suicidal ideation is presented in Figure 1. The exogenous variables in the model included hospitalization of a close family member due to COVID-19 and the perceived impact of pandemic-related measures on five different life domains. Subjective wellbeing, anxiety symptoms, and life satisfaction were specified as mediating variables, with subjective wellbeing and anxiety modeled as latent constructs and life satisfaction included as an observed variable. The presence of suicidal ideation was included as an observed outcome variable. Gender, age, family financial wellbeing, and family structure were included as covariates because previous research has consistently demonstrated their associations with adolescent mental health and suicidality.

## 3 Results

### 3.1 Descriptive statistics and bivariate associations between variables

Suicidal ideation was reported by 16.7% of adolescents. The mean score for subjective wellbeing was 50.91 (SD = 23.91), for anxiety 9.70 (SD = 7.72), and for life satisfaction 7.34 (SD = 3.66). A total of 10.9% of adolescents reported that a close family member had been hospitalized due to COVID-19. The mean perceived impact of the pandemic on family relationships was 3.58 (SD = 1.08), on friendships 3.58 (SD = 1.06), on academic performance 3.33 (SD = 1.08), on future expectations 3.27 (SD = 1.09), and on family financial circumstances 3.51 (SD = 1.01), where 5 indicated a very positive impact and 1 indicated a very negative impact.

Adolescents reporting suicidal ideation had significantly lower levels of subjective wellbeing and life satisfaction and significantly higher levels of anxiety than those without suicidal ideation (Table 1). Effect sizes were large. Furthermore, adolescents reporting suicidal ideation perceived the impact of the pandemic less favorably across all assessed life domains and were more likely to report the hospitalization of a close family member due to COVID-19. Effect sizes ranged from small (hospitalization of a family member) to medium (perceived pandemic-related experiences).

**Table 1:** *T*-tests for independent samples and chi-square tests between suicidal thoughts, mental health, and pandemic experiences.

	Suicidal ideation						*t*	*df*	*d*
	Yes			No					
	*N*	*M*	*SD*	*N*	*M*	*SD*			
Wellbeing	1,388	31.77	21.37	6,790	54.82	22.48	36.283***	2063.7	1.03
Anxiety	1,404	17.03	7.94	6,892	8.21	6.76	−38.859***	1840.3	1.26
Life satisfaction	1,418	5.72	2.23	6,944	7.65	1.66	30.765***	1748.9	1.09
Impact on family relationships	1,397	2.97	1.15	6,861	3.70	1.02	21.991***	1865.7	0.70
Impact on relationships with friends	1,395	3.29	1.10	6,870	3.64	1.04	10.918***	1935	0.33
Impact on school performance	1,401	2.90	1.17	6,895	3.42	1.04	15.392***	1871.1	0.49
Impact on future expectations	1,401	2.71	1.15	6,882	3.38	1.04	20.278***	1889	0.63
Impact on the family’s financial situation	1,389	3.16	1.05	6,853	3.58	0.99	13.676***	1914.6	0.42

Hospitalization of a family member	*Yes*			*No*					
	*N*	*%*		*N*	*%*		*x* ^2^	*df*	*V*
Yes	226	16.47		655	9.71		53.868***	1	0.08
No	1,146	83.53		6,090	90.29				

Due to the violation of the assumption of equal variances, *t*-tests adjusted for unequal variances are reported. *d*, Cohen’s d; *V*, Cramer’s V, N, Sample Size; M, Mean; SD, Standard Deviation. ***p < 0.001.

All correlations between pandemic experiences and mental health indicators were statistically significant (*p* < 0.01) (Table 2). Anxiety was associated with more negative pandemic experiences related to family relationships, friendships, academic performance, and future expectations, whereas subjective wellbeing and life satisfaction were associated with more positive experiences in these domains.

**Table 2:** Correlation coefficients between pandemic experiences and mental health.

Perceived impact on…	Anxiety	Wellbeing	Life satisfaction
…family relationships	−0.315**	0.354**	0.347**
…relationships with friends	−0.230**	0.255**	0.198**
…school performance	−0.225**	0.277**	0.232**
…future expectations	−0.312**	0.380**	0.325**
…the family finances	−0.216**	0.266**	0.261**

**p < 0.01.

**Figure 2:**
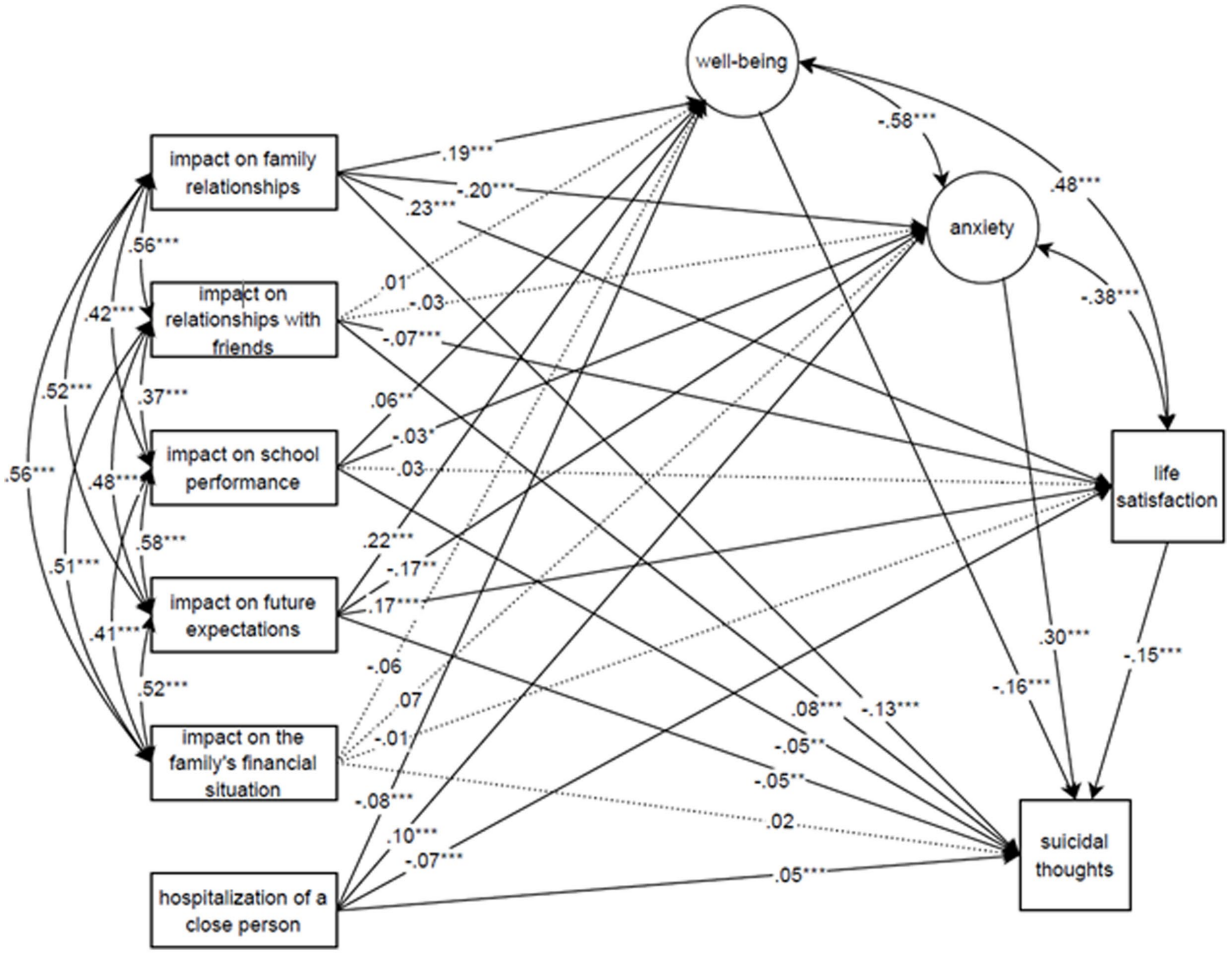
Model showing relationships between COVID-19 pandemic experiences, subjective wellbeing, anxiety symptoms, life satisfaction, and suicidal ideation. The model shows standardized effects. Regression coefficients are linear for continuous dependent variables (subjective wellbeing, anxiety, life satisfaction) and probit for the binary dependent variable (suicidal ideation). Covariates (gender, age, family financial wellbeing, and family type) and manifest variables in subjective wellbeing and anxiety are not displayed for clarity. Dashed lines indicate non-significant associations; solid lines indicate statistically significant associations. *p < 0.05, **p < 0.01, ***p < 0.001.

The proposed model, in which pandemic experiences were associated with suicidal ideation both directly and indirectly through mental health indicators (Figure 2), demonstrated good model fit, χ^2^(204) = 519.818, RMSEA = 0.014, CFI = 0.980, TLI = 0.967, SRMR = 0.025. The model explained 27.9% of the variance in subjective wellbeing, 21.4% in anxiety symptoms, 19.7% in life satisfaction, and 45.4% in suicidal ideation. Correlations among the five pandemic-experience variables ranged from 0.41 to 0.58 (*p* < 0.001).

The absence of a close family member’s hospitalization due to COVID-19, as well as more positively perceived impacts of the pandemic on family relationships and future expectations, were associated with higher levels of subjective wellbeing and life satisfaction (see Figure 2 and Supplementary Table S1 for all standardized path coefficients and standard errors). A less favorably perceived impact on friendships was positively associated with life satisfaction. Less favorable perceived impacts on family relationships, academic performance, and future expectations, as well as the hospitalization of a close family member due to COVID-19, were associated with higher levels of anxiety.

More negatively perceived impacts on family relationships, academic performance, and future expectations, as well as more positively perceived impacts on friendships and the hospitalization of a close family member due to COVID-19, were associated with higher levels of suicidal ideation. Perceived impacts on family financial circumstances were not significantly associated with any of the mental health indicators or with suicidal ideation. Finally, lower subjective wellbeing, lower life satisfaction, and higher anxiety were significantly associated with suicidal ideation, with the strongest association observed for anxiety.

Pandemic measures affecting family relationships showed the largest total association with suicidal ideation (Table 3). The associations between pandemic-related impacts on family relationships, future expectations, and the hospitalization of a family member and suicidal ideation were partially mediated by all three mental health indicators. The association between the perceived impact on friendships and suicidal ideation was mediated by life satisfaction, while the association between perceived academic impacts and suicidal ideation was mediated by subjective wellbeing and anxiety.

**Table 3:** Standardized total and indirect effects of pandemic experiences on suicidal ideation through mental health measures.

Total effects on suicidal ideation	Standardized effect	*SE*	*p*	95% CI	
				LL	UL
Family relationships	−0.259	0.028	<0.001	−0.314	−0.204
Relationships with friends	0.078	0.029	<0.001	0.022	0.134
School performance	−0.066	0.021	0.002	−0.107	−0.025
Future expectations	−0.170	0.041	<0.001	−0.251	−0.088
The family finances	0.060	0.051	0.236	−0.039	0.159
Hospitalization of a family member	0.104	0.016	<0.001	0.079	0.135
Indirect effects on suicidal ideation through life satisfaction					
Family relationships	−0.034	0.004	<0.001	−0.042	−0.026
Relationships with friends	0.011	0.002	<0.001	0.007	0.015
School performance	−0.004	0.003	0.181	−0.009	0.002
Future expectations	−0.026	0.004	<0.001	−0.058	−0.018
The family finances	0.001	0.010	0.918	−0.019	0.021
Hospitalization of a family member	0.010	0.002	<0.001	0.007	0.014
Indirect effects on suicidal ideation through wellbeing (WHO-5)					
Family Relationships	−0.032	0.006	<0.001	−0.049	−0.020
Relationships with friends	−0.001	0.003	0.711	−0.007	0.005
School performance	−0.010	0.004	0.011	−0.018	−0.002
Future expectations	−0.038	0.010	<0.001	−0.058	−0.018
The family finances	0.010	0.013	0.443	−0.016	0.036
Hospitalization of a family member	0.013	0.003	<0.001	0.007	0.019
Indirect effects on suicidal ideation through anxiety (GAD-7)					
Family Relationships	−0.062	0.014	<0.001	−0.090	−0.034
Relationships with friends	−0.009	0.010	0.352	−0.008	0.010
School performance	−0.009	0.004	0.035	−0.018	−0.001
Future expectations	−0.052	0.026	0.042	−0.102	−0.002
The family finances	0.022	0.022	0.300	−0.020	0.065
Hospitalization of a family member	0.030	0.006	<0.001	0.018	0.042

LL, lower limit of the confidence interval; UL, upper limit of the confidence interval.

Indirect associations of pandemic experiences with suicidal ideation through life satisfaction accounted for 1.7–14.1% of total effects, through subjective wellbeing from 1.3 to 22.4%, and through anxiety from 11.5 to 36.7%.

## 4 Discussion

We examined associations between experiences during the COVID-19 pandemic and suicidal ideation among Slovenian adolescents, both directly and indirectly through life satisfaction, subjective wellbeing, and anxiety. The observed prevalence of suicidal ideation in our study was similar to that reported in China (50) and lower than the prevalence reported in other studies (6, 9, 51), likely due to differences in the defined study periods (e.g., autumn 2021 vs. the period from 2020 to February 2021). All examined pandemic-related experiences explained a significant proportion of variance in suicidal ideation, except for the perceived impact of the pandemic on family finances. Given the cross-sectional nature of our data, we cannot infer causality. The strongest overall associations with suicidal ideation were observed for the perceived impact of pandemic measures on family relationships (negative association) and the hospitalization of a family member (positive association).

Previous research has shown a positive correlation between home relationships during COVID-19 confinement and adolescent mental health through an indirect effect of positive affect (52). Family support has also been shown to reduce the sense of loneliness and mitigate depressive symptoms during the lockdown (53). Furthermore, improvement in parent–child relationships during school suspension encouraged adolescents to use more positive emotional regulation strategies (54).

The hospitalization of a close family member was linked to increased anxiety, lower wellbeing, and reduced life satisfaction. Similarly, Elharake et al. (55) found that having a family member or friend who was a healthcare worker or knowing someone infected with COVID-19 was strongly associated with worse mental health outcomes, particularly anxiety in children and adolescents.

The perceived impact of the pandemic on future expectations, school performance, and friendships was also significantly associated with suicidal ideation. The first two domains were negatively related to suicidal ideation, whereas, somewhat unexpectedly, a more positive perceived impact on friendships was associated with higher levels of suicidal ideation. While the bivariate correlations showed the expected association between greater disruption of friendships during the pandemic and higher suicidal ideation, the SEM revealed an opposite direction of association once other variables were controlled for. This finding should be interpreted with caution. Collinearity diagnostics indicated that variance inflation factors were within acceptable limits (VIF < 1.95), suggesting that problematic multicollinearity was unlikely. However, moderate-to-strong correlations among the pandemic-related predictors (*r* = 0.37–0.58) indicated substantial shared variance. Therefore, the reversed path may reflect a statistical suppression effect arising from overlapping variance among the predictors rather than a straightforward substantive association, and further research is needed to clarify the underlying mechanisms.

The perceived impact of the pandemic on school performance was negatively related to suicidal ideation, suggesting that adolescents who evaluated their academic experience during the pandemic more positively reported lower levels of suicidal ideation. Previous research suggests that the COVID-19 pandemic might not have significantly affected suicide rates among children and adolescents during periods of school closure (56), and that school closures may even have temporarily reduced suicidal ideation and behavior, as well as the incidence of parental psychological and physical abuse, by allowing families to spend more time together (28). However, other studies (30) reported that the most common stressors among youth whose suicides were linked to the COVID-19 pandemic included isolation, school failure, and other school-related problems.

The perceived impact on the family’s financial situation was not significantly related to suicidal ideation or to other mental health indicators. This finding may suggest that other factors are more closely associated with suicidal ideation in adolescence, as stated in previous research among Slovene adolescents, where the most important predictors of suicidal ideation were loneliness, depressive feelings, and lower family support (18). However, perceiving the family to be not well off financially appeared to increase the probability of suicidal behaviors among adolescents in a study conducted before the pandemic (57). In addition, low family income and income loss were found to increase anxiety and depression levels among children and adolescents exposed to the COVID-19 pandemic (58, 59).

These findings can be interpreted within the frameworks of the Conservation of Resources (COR) theory (32) and the Dual-Factor Model of Mental Health (DFM) (33). In line with COR theory, adolescents who perceived the pandemic as having a negative impact on key life domains (e.g., family relationships, school, future expectations) experienced a loss of important personal and social resources, reflected in lower wellbeing and higher anxiety, which increased their risk for suicidal ideation. Conversely, positive family relationships appeared to function as protective resource gains. Consistent with the DFM, both negative (anxiety) and positive (wellbeing, life satisfaction) aspects of mental health showed unique associations with suicidal ideation, highlighting that preventing distress alone is insufficient without also strengthening positive psychological functioning. Together, these frameworks suggest that preventive efforts should aim not only to reduce psychological distress but also to enhance adolescents’ personal and relational resources that support resilience. This theoretical perspective offers a comprehensive explanation of how stressors related to resource loss and the dual dimensions of mental health jointly shape adolescents’ vulnerability to suicidality during large-scale crises such as the COVID-19 pandemic. Furthermore, as shown by Peng et al. (60), post-pandemic mental health recovery trajectories are influenced by gender, grade, sibling status and parenting styles as well as the number and the severity of mental health problems. These findings further underscore the importance of targeted interventions to support adolescent mental health recovery following large-scale public health crises.

Beyond these theoretical frameworks, our findings are also consistent with the broader perspective proposed by the Lancet Psychiatry Commission on Youth Mental Health, which argues that adolescent mental health should be understood within the context of interacting societal megatrends rather than solely as an individual phenomenon (1). According to the Commission, large-scale crises—including the COVID-19 pandemic, widening social inequalities, economic insecurity, digital transformation, and other global disruptions—can undermine key developmental resources and increase vulnerability to mental health problems (1). Our findings support this perspective by demonstrating that adolescents’ perceptions of disruptions in family relationships, future expectations, school experiences, and family member hospitalization were associated with suicidal ideation through both positive and negative dimensions of mental health.

Suicide prevention requires integrated approaches addressing emotional, social, and psychological dimensions rather than focusing solely on suicidal behavior (61). Schools represent a key setting for early identification and intervention, offering opportunities for systematic screening and the implementation of evidence-based mental health promotion approaches.

Adolescence is increasingly recognized as a critical developmental period for implementing mental health promotion and suicide prevention initiatives. Mental health difficulties and suicidal thoughts frequently emerge during this stage, reflecting the significant psychological, social, and neurobiological changes associated with adolescent development. From a public health perspective, early intervention is particularly important, as many mental health conditions begin before adulthood and timely support can reduce the risk of persistent psychological difficulties, self-harm, and suicidal behavior later in life. Accordingly, international organizations such as the World Health Organization (WHO) and the European Commission identify prevention, early detection, and intervention among children and adolescents as key priorities for contemporary mental health and suicide prevention strategies (4, 20, 62). Our findings suggest that preventive efforts may benefit from extending beyond the individual (micro) level to include the family (meso) level as a critical target for action. More broadly, they support growing international calls to address adolescent mental health through integrated public health approaches that consider the wider social, economic, educational, and environmental determinants, social inequalities shaping young people’s development (1). In addition, the findings support the value of population-based monitoring systems, such as the HBSC study, in capturing both social and emotional determinants of adolescent mental health and informing timely public health responses.

### 4.1 Study limitations and strengths

As a major strength, this study is based on a large nationally representative sample of Slovenian adolescents, which is particularly important given that the prevalence of suicidality, anxiety, and depression may vary across regions. Moreover, the study integrated both positive and negative mental health indicators within the same model, combining life satisfaction and subjective wellbeing with anxiety. This is particularly valuable because it extends the focus beyond psychological distress alone and aligns with the Dual-Factor Model, which emphasizes the importance of considering both positive and negative dimensions when conceptualizing and measuring mental health. Furthermore, distinguishing between subjective wellbeing and life satisfaction represents an additional strength of the study. Specifically, this approach captures both the emotional dimension reflected by the WHO-5 and the cognitive evaluation reflected by life satisfaction, thereby providing a more comprehensive operationalization of positive functioning within the DFM framework.

However, several limitations should be acknowledged. First, all data were self-reported, which may introduce social desirability bias and subjective misinterpretation of items, particularly those concerning sensitive topics such as mental health and suicidal ideation. Self-report measures are also susceptible to recall bias, as adolescents may not accurately remember or may reinterpret their experiences over the course of the pandemic. In addition, suicidal ideation was assessed using a single dichotomous (yes/no) item, which limits a more comprehensive understanding of suicide risk. A positive response indicates the presence of serious suicidal thoughts but does not capture their severity, frequency, persistence, suicidal intent, or co-occurring self-harm behaviors, all of which are important for understanding the complexity of the suicidal process during adolescence. However, developmental research suggests that suicidal thoughts become increasingly common during adolescence, reflecting the unique psychological, social, and neurobiological changes associated with this developmental stage. While suicidal ideation should not be considered normative, its prevalence increases substantially during early and mid-adolescence, making this period a critical window for prevention and early intervention (63).

Second, the study is cross-sectional, limiting the ability to establish causal relationships between pandemic-related experiences, mental health indicators, and suicidal ideation. The observed associations therefore reflect correlations rather than directional effects. Furthermore, although the structural equation model identified indirect associations consistent with the proposed mediation framework, the cross-sectional nature of the data precludes conclusions regarding causal mechanisms or temporal ordering. Accordingly, the identified mediation pathways should be interpreted as hypothetical and model-consistent rather than as evidence of causal mediation.

Third, the study lacks baseline pre-pandemic data, which prevents direct comparisons of mental health and suicidality before and during the pandemic. The absence of longitudinal reference data limits our ability to determine whether the observed patterns reflect pandemic-specific experiences or pre-existing trends in adolescent mental health and wellbeing. In addition, although we included a broad range of contextual and relational factors, other potentially important variables—such as pre-existing mental health conditions, genetic predispositions, or unmeasured environmental stressors—were not assessed and may have influenced the observed associations.

Finally, because the data were collected during the pandemic, the findings do not provide information about the long-term consequences of these experiences for adolescent mental health or about potential post-pandemic recovery trajectories.

## 5 Conclusion

The study found that adolescents’ experiences during the COVID-19 pandemic were significantly associated with mental health and suicidal ideation. Lower levels of wellbeing and life satisfaction, together with higher levels of anxiety, were significantly associated with suicidal ideation, with anxiety showing the strongest association. Among the pandemic-related experiences examined, perceived changes in family relationships showed the strongest overall association with suicidal ideation, highlighting the potential importance of family dynamics for adolescent mental health during periods of major societal disruption.

These findings suggest that integrated family- and school-based public health approaches may be valuable for supporting adolescent mental health during crises. Such approaches may include strengthening family support systems, early identification of anxiety, and the implementation of school-based programs that promote wellbeing, social connectedness, and positive future orientation.

The findings may help inform policymakers seeking to mitigate the mental health consequences of future public health emergencies. Beyond the COVID-19 context, the results may also provide insights relevant to preparedness planning for future crises. Longitudinal research is needed to clarify the temporal ordering and directionality of the observed associations.

## Data Availability

The data analyzed in this study is subject to the following licenses/restrictions: The datasets analyzed in this study are available from the corresponding author or through the HBSC data access protocol upon reasonable request. Requests to access these datasets should be directed to helena.jericek@nijz.si or statisticna.pisarna@nijz.si.
