# Supplementary material for: Associations between COVID-19 pandemic experiences and suicidal ideation among adolescents: the mediating role of life satisfaction, subjective wellbeing, and anxiety
Source: Front Public Health. 2026 Aug 5;14:1834645. doi: 10.3389/fpubh.2026.1834645 (PMC13487744; doi:10.3389/fpubh.2026.1834645)
Supplement: Supplementary file 1 [file Table_1.DOCX]

**Supplementary Material**

**Supplementary Table**

**Table S1:** Standardized Direct Effects of the Structural Equation Model

| **Direct Path (Predictor → Outcome)** | *Standardized Effect* | *SE* | *p* |
| --- | --- | --- | --- |
| **Paths to Subjective Well-being (WHO-5)** | | | |
| Family relationships → Well-being | 0.193 | 0.016 | <.001 |
| Relationships with friends → Well-being | 0.007 | 0.018 | .705 |
| School performance → Well-being | 0.059 | 0.018 | .001 |
| Future expectations → Well-being | 0.224 | 0.036 | <.001 |
| Family finances → Well-being | -0.060 | 0.074 | .418 |
| Hospitalization of a family member → Well-being | -0.076 | 0.012 | <.001 |
| **Paths to Anxiety (GAD-7)** | | | |
| Family relationships → Anxiety | -0.202 | 0.018 | <.001 |
| Relationships with friends → Anxiety | -0.030 | 0.028 | .287 |
| School performance → Anxiety | -0.030 | 0.012 | .016 |
| Future expectations → Anxiety | -0.171 | 0.058 | .003 |
| Family finances → Anxiety | 0.074 | 0.060 | .222 |
| Hospitalization of a family member → Anxiety | 0.098 | 0.011 | <.001 |
| **Paths to Life Satisfaction** | | | |
| Family relationships → Life Satisfaction | 0.234 | 0.012 | <.001 |
| Relationships with friends → Life Satisfaction | -0.073 | 0.012 | <.001 |
| School performance → Life Satisfaction | 0.025 | 0.019 | .176 |
| Future expectations → Life Satisfaction | 0.175 | 0.015 | <.001 |
| Family finances → Life Satisfaction | -0.007 | 0.070 | .918 |
| Hospitalization of a family member → Life Satisfaction | -0.071 | 0.011 | <.001 |
| **Paths to Suicidal Ideation** | | | |
| Well-being (WHO-5) → Suicidal Ideation | -0.168 | 0.028 | <.001 |
| Anxiety (GAD-7) → Suicidal Ideation | 0.306 | 0.050 | <.001 |
| Life Satisfaction → Suicidal Ideation | -0.147 | 0.016 | <.001 |
| Family relationships → Suicidal Ideation | -0.130 | 0.021 | <.001 |
| Relationships with friends → Suicidal Ideation | 0.078 | 0.022 | .001 |
| School performance → Suicidal Ideation | -0.043 | 0.018 | .015 |
| Future expectations → Suicidal Ideation | -0.054 | 0.020 | .008 |
| Family finances → Suicidal Ideation | 0.026 | 0.023 | .242 |
| Hospitalization of a family member → Suicidal Ideation | 0.051 | 0.015 | .001 |
